# Regulation of the tumor immune microenvironment by cancer-derived circular RNAs

**DOI:** 10.1038/s41419-023-05647-w

**Published:** 2023-02-16

**Authors:** Liping Guan, Qian Hao, Fenfen Shi, Bo Gao, Mengxin Wang, Xiang Zhou, Tao Han, Wenjie Ren

**Affiliations:** 1 School of Basic Medical Sciences, Xinxiang Medical University, Xinxiang, 453003 China; 2 Institutes of Health Central Plains, Xinxiang Medical University, Xinxiang, 453003 China; 3 Fudan University Shanghai Cancer Center and Institutes of Biomedical Sciences, Fudan University, Shanghai, 200032 China; 4 Department of Oncology, Shanghai Medical College, Fudan University, Shanghai, 200032 China; 5 Key Laboratory of Breast Cancer in Shanghai, Fudan University Shanghai Cancer Center, Fudan University, Shanghai, 200032 China; 6 Shanghai Key Laboratory of Medical Epigenetics, International Co-laboratory of Medical Epigenetics and Metabolism (Ministry of Science and Technology), Institutes of Biomedical Sciences, Fudan University, Shanghai, 200032 China

**Keywords:** Cancer microenvironment, Immunotherapy

## Abstract

Circular RNA (circRNAs) is a covalently closed circular non-coding RNA formed by reverse back-splicing from precursor messenger RNA. It is found widely in eukaryotic cells and can be released to the surrounding environment and captured by other cell types. This, circRNAs serve as connections between different cell types for the mediation of multiple signaling pathways. CircRNAs reshape the tumor microenvironment (TME), a key factor involved in all stages of cancer development, by regulating epithelial-stromal transformation, tumor vascularization, immune cell function, and inflammatory responses. Immune cells are the most abundant cellular TME components, and they have profound toxicity to cancer cells. This review summarizes circRNA regulation of immune cells, including T cells, natural killer cells, and macrophages; highlights the impact of circRNAs on tumor progression, treatment, and prognosis; and indicates new targets for tumor immunotherapy.

## Facts

- The expression of circRNAs is frequently dysregulated in human cancers.
- CircRNAs play different roles during tumorigenesis and cancer progression.
- CircRNAs regulate T cells, NK cells, and macrophages to reshape the tumor microenvironment.
- CircRNA regulation of the tumor microenvironment provides potential therapeutic opportunities for cancer treatment.

## Questions

- Why circRNAs have multiple functions in the same or different human cancers. What are the underlining molecular determinants of this specificity?
- Does dose-dependent targeting of circRNAs work in mouse models, at least in three-dimensional tumor organoid models?
- Is circRNA targeting applicable in clinical trials?
- Can we design prophylactic or therapeutic anti-cancer approaches based on genetic of polymorphisms of circRNAs?

## Introduction

Circular RNAs (circRNA) is a closed circular molecule that is resistant to exonucleases, and is thus stable and widespread in animals and plants. CircRNA was discovered in 1976 when the Sanger team studied virus-like RNAs [1]. In 1991, Nigro et al. [2] accidentally discovered a normal novel RNA product. Due to its low expression and the limitations of detection technology, circRNA was originally considered to be an aberrant product of RNA splicing. Recently, with advances in high-throughput sequencing technology, increasing numbers of circRNAs have been characterized and their roles and mechanisms have become active areas of investigation [3–5].

The immune system maintains homeostasis through immunomodulation, surveillance, and the prevention of pathogen invasion. The immune response coordinates a variety of immune cells and has antiviral, antibacterial, and antitumor functions. With rapid developments in oncology, immunology, molecular biology, and related disciplines, immunotherapies such as immune checkpoint inhibitors, tumor vaccines, and adoptive cell therapy have revolutionized cancer treatment. However, therapeutic responses, especially those of solid tumors, have been unsatisfactory in clinical trials and clinical applications. Recent studies have demonstrated that circRNAs are involved in cancer development [6–8] and immune responses [9–12]. In this review, we discuss the roles of circRNAs in the regulation of immune cells, immune-related molecules, and tumor immunity. We anticipate that this summary of current knowledge will facilitate the development of strategies to target circRNAs in the immune microenvironments of human cancers.

## Biogenesis and function of circRNAs

CircRNA is a class of non-coding RNA generated from precursor messenger RNA (mRNA). Most circRNAs originate from exons in gene coding regions; others originate from 3′–untranslated regions (UTRs), 5'-UTRs, introns, intergenic regions, and antisense RNA [13, 14]. CircRNAs can be divided into four categories based on their sequence origin: (1) exonic circular RNAs (EciRNAs) derived from exons of the parent gene; (2) lasso-type or circular intronic RNAs (ciRNAs) derived from introns; (3) exonic–intronic circular RNAs (EIciRNAs) derived from both exons and introns; and (4) other circRNAs, including those derived from antisense strand transcripts (antisense circRNAs) and those derived from intergenic sequences or other unannotated genomic sequences (intergenic circRNAs) [15]. About 80% of circRNAs are EciRNAs localized mainly to the cytoplasm, whereas ciRNAs and EIciRNAs are often localized to the nucleus. CircRNAs are relatively evolutionarily conserved in different species. Jeck et al. [16] used the genome-wide RNase R enrichment method to detect >25,000 circRNAs in fibroblasts. Wang et al. [17] observed circRNA expression in fungi, plants, and prokaryotes, reflecting a high degree of conservation and widespread distribution among species. The expression of the same circRNA varies greatly under diseased and non-diseased conditions, among tissues, and during different time periods. The half-life of circRNAs exceeds that of the associated linear mRNA, as the covalent closed-loop structure lacks 5′ and 3′ends, which makes circRNAs more resistant to the exonuclease RNase R [18].

**Fig. 1:**
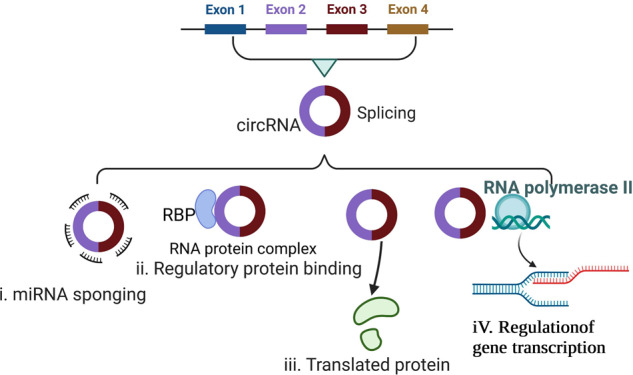
Biological functions of circRNAs. (i) Serving as molecular sponges for miRNA. (ii) Regulating protein binding. (iii) Encoding protein. (iv) Regulating gene transcription. RBP RNA-binding proteins.

CircRNAs have four main biological functions (Fig. 1). (1) As they contain a large number of micro-RNA (miRNA) binding sites, they serve as molecular sponges and compete for miRNA binding to target mRNAs, thereby upregulating the expression of target genes [19–22]. (2) They participate in regulatory protein binding. Various RNA-binding proteins (RBPs) play crucial roles in RNA splicing, RNA stabilization, and mRNA translation. They bind to RNA and facilitate its processing and translation. CircRNAs interact with RBPs to form an RNA-protein complex, affecting RBP-mediated gene expression [23–25]. (3) They participate in protein encoding, as some circRNAs can be translated into peptides by ribosomes [26, 27]. (4) They regulate gene transcription, promoting parental gene expression by interacting with U1 small ribonucleoprotein or enhancing RNA polymerase activity [28, 29].

## circRNAs regulate T cells

T cells play an important role in the antitumor immune response [30]. T-cell activation is initiated through interaction with antigenic ligands, which are short peptide fragments bound to major histocompatibility complex class I (MHC I) and class II (MHC II) molecules. CD4^+^ T cells recognize mainly exogenous antigens presented by MHC II molecules, whereas CD8^+^ T cells recognize mainly endogenous antigens presented by MHC I molecules [31, 32]. Endogenous tumor antigens are processed intracellularly into antigenic peptides, and CD8^+^ T cells are activated upon recognizing antigenic peptide–MHC I complexes on tumor cell surfaces; these activated cells kill tumor cells by secreting perforin and granzymes, tumor necrosis factor, and lymphatic toxins [33]. They also kill tumor cells directly through apoptotic signals by interacting with human factor–related apoptosis and its ligand [34, 35]. Soluble antigens secreted by tumor cells are presented to CD4^+^ T cells by antigen-presenting cells, activating the CD4^+^ T cells. Primed CD4^+^ T cells activate B and CD8^+^ T cells to kill tumor cells [36, 37].

**Fig. 2:**
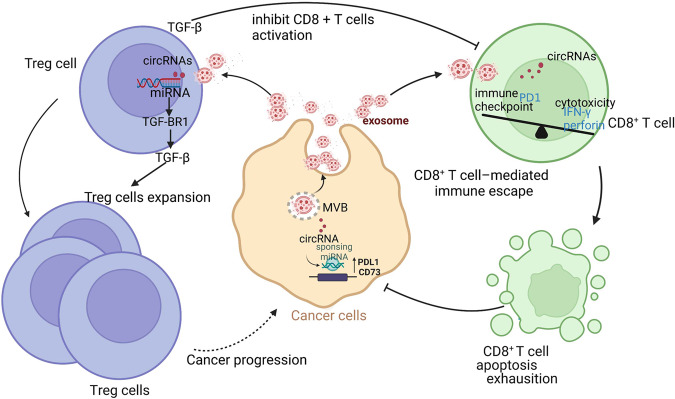
Circular RNAs modulate T-cell function. TGF-β transforming growth factor-β. TGF-βR I transforming growth factor beta receptor I. IFN-γ interferon γ. Treg regulatory T cells. PD1 programmed cell death. PDL1 programmed cell death ligand 1.

Mounting evidence indicates that tumor cells secrete exosomes into the circulation, which deliver certain intracellular components, such as circRNAs, into the tumor microenvironment (TME), reshaping it [38–40]. Tumor cell-derived circRNAs have recently been reported to play a vital and direct role in tumor immune escape (Table 1). Mechanically, circRNAs enhance the interaction between the immunosuppressive molecule programmed death receptor 1 (PD-1) and its ligand (PD-L1) by upregulating PD-1 expression in T cells, suppressing T-cell activation and cytokine secretion. Exosomes derived from different tumor cells deliver various circRNAs to T cells to inhibit their killing ability via PD-1 upregulation. Those derived from ovarian cancer cells were found to deliver circ-0001068 into T cells, increasing PD-1 expression via miR-28-5p sponging and thereby causing T-cell exhaustion [41]. In lung adenocarcinoma, circRNA-002178 was found to enter CD8^+^ T cells via exosomes and upregulate PD-1 expression by absorbing miR-34a [42]. circRNA can also upregulate the expression of the immune checkpoint molecules PD-L1 and CD73 on tumor cell surfaces via miRNA sponging, which helps tumor cells to escape recognition and death by T cells. Multiple studies have shown that circRNAs regulate PD-L1 expression via the circRNA–miRNA–mRNA axis, for instance, the circRNA of vimentin, CDR1-AS, hsa_circ_0003288, hsa_circ_0000190, hsa_circ_0046523, circ-CPA4, hsa-circRNA-002178, circ_0000284, circ_001678, circ-HSP90A, and circIGF2BP3 (Table 1) [43–54]. Mechanistically, they upregulate PD-L1 expression by sponging miRNAs in tumor cells, which induces T-cell apoptosis and immune escape (Fig. 2). A recent study showed that circ_0136666 induces regulatory T (Treg) cell activation by increasing PD-L1 expression through miR-497, leading to the immune escape of colorectal cancer (CRC) cells. Preclinical studies have shown that the upregulation of CD73, believed to be a novel immune checkpoint molecule, promotes tumor growth and disease progression by TME remodeling [55]. The inhibition of CD73 may promote the activity of T cells and other immune cells, enhancing antitumor immune surveillance via the adenosine pathway [56]. Xu and colleagues found that the expression of circHMGCS1-016 was upregulated in intrahepatic cholangiocarcinoma tissue, and that this upregulation correlated with poor survival; CD73 and GAL-8 were also upregulated in this tissue. Mechanistically, circHMGCS1-016 induced CD73 and GAL-8 expression by sequestering miR-1236-3p [57].

**Table 1:** circRNAs regulate T cells.

circRNA	T cells	Targets/pathways	regulation of immune responses	Cancer	References
circ-0001068	T cell	circ-0001068 induce PD1 expression and T cell exhaustion by sponging miR-28-5p	prompt T cell exhaustion	ovarian cancer	[41]
CircRNA-002178	CD8^+^T cell	CircRNA-002178/miR-28-5p /PD-L1 in CD8^+^T cell induce T-cell exhaustion	induce T-cell exhaustion	lung adenocarcinoma	[42]
hsa_circ_0136666	Treg cell	hsa_circ_0136666 /miR-497/ PD-L1 b inducing the activation of Treg cells and leading to the immune escape	inducing the activation of Treg cell	colorectal cancer	[43]
circ-VIM	CD8^+^T cell	circ-VIM / miR-124/PD-L1 in CD8^+^T cell induces immunity escape	damage the viability and cytotoxicity of CD8^+^ T cells	esophageal cancer	[44]
hsa_circ_0000190	T cell	hsa_circ_0000190 /PD-L1 mRNA/soluble PD-L1 (sPD-L1) interfering with T-cell activation induces immunity escape	interfering with T-cell activation,	Non-Small-Cell Lung Cancer	[47]
Hsa_circ_0046523	CD4^+^T cell CD8^+^T cell Treg cell	circ_0046523/miR-148a-3p/PD-L1 axis mediates immunosuppressive microenvironment	decrease the proportion of CD4 + and CD8 + T cells, and increase the proportion of Tregs; promoted the apoptosis and exhaustion of CD8 + T cell, inhibited CD8 + T cell function	pancreatic cancer	[48]
circ_001678	CD8^+^T cell	circ_001678 /miR-326/ZEB1 /PD-1/PD-L1/axis inducing immune escape	promote CD8^+^ T cell apoptosis; decrease the percentage of CD8^+^ T cells	non-small cell lung cancer	[51]
Circ-HSP90A	CD8^+^ T cell	circ-HSP90A promoted CD8 + T cells apoptosis via upregulating PD-L1 expression	induce CD8 + T cell apoptosis	non-small cell lung cancer	[52]
hsa_circ_0079587 (circIGF2BP3)	CD8^+^T cell	hsa_circ_0079587/PXP3/PD-L1 axis inducing immune escape	inducing the inactivity and exhaustion of T cells	non-small-cell lung cancer	[53]
CircKRT1	CD8^+^ T cell	circKRT1/miR-495-3p/PDL1 axis induces immune evasion	weaken CD8 + T cell cytotoxicity and induce CD8 + T cell apoptosis	oral squamous cell carcinoma	[54]
circHMGCS1–016	CD4^+^T cell CD8^+^T cell	circHMGCS1–016/miR-1236-3p/CD73 and GAL-8 axis induces immunosuppression	Number of T cell decrease and function of T cell damaged	intrahepatic cholangiocarcinoma	[57]
circ_002172	CD8^+^T cell	circ_002172/miR-296-5p/CXCL12 axis inducing immune escape	inhibits cytotoxic T lymphocytes (CTL) infiltration	breast cancer	[58]
Circ_0008287	CD8 ^+^ T cell	circ_0008287/miR-548c-3p/CLIC1 axis	damage the function of CD8^+^ T cell and induce apoptosis	gastric cancer	[64]
circTRPS1	CD8^+^ T cell	circTRPS1/miR141-3p/GLS1 axis CD8^+^ T cell inducing exhaustion	CD8^+^ T cell exhaustion	bladder cancer	[65]
has_circ_0069313	Treg cell	has_circ_0069313 /miR-325-3p/Foxp3 axis in Treg cell induces immunity escape	promotes Treg function	oral squamous cell carcinoma	[69]
circGSE1	Treg cell CD8^+^ T	circGSE1/miR-324-5p/TGFBR1/ Smad3 axis inducing Tregs expansion	Tregs suppress the function of CD8^+^ T cell	hepatocellular carcinoma	[70]

CircRNAs modulate antitumor T-cell activity through various mechanisms. In addition to regulating immune checkpoint molecules, circ_002172 inhibited cytotoxic T cell (CTL) infiltration and promoted breast cancer development by upregulating C-X-C motif chemokine ligand 12 (CXCL12) expression via miR-296-5p [58]. CXCL12 upregulation promotes tumor growth and leads to the recruitment of immunosuppressive cells to prevent CTL infiltration of tumors [59]. The dysfunctional expression of chloride intracellular channel 1 (CLIC1), a member of the chloride channel protein family, is related closely to tumor invasion, metastasis, treatment resistance, and prognosis [60]. High CLIC1 levels have been found in a variety of malignant tumors, including cervical cancer, breast cancer, hepatocellular carcinoma, gastric cancer, gallbladder carcinoma, and CRC [61–63]. Li et al. [64] found that circ_0008287 promotes the immune escape of gastric cancer cells by impairing miR-548c-3p–dependent CLIC1 inhibition. CLIC1 depletion in these cells suppresses CD8^+^ T-cell apoptosis, thereby increasing interferon (IFN)-γ secretion. CircRNAs have been demonstrated to suppress CD8^+^ T-cell activity by regulating tumor cell metabolism. Glutamine metabolism is partly responsible for the detoxification of reactive oxygen species, which profoundly influences the TME. circTRPS1 was recently reported to promote the malignant phenotype of bladder cancer (BCa) and CD8^+^ T-cell exhaustion therein. Mechanistically, it regulates redox equilibrium by altering glutaminase 1–mediated glutamine metabolism [65]. circRNAs have also been found to affect glutamate, glucose, and lipid metabolism, among others [66, 67].

Treg cells are subsets of T lymphocytes that mediate immune suppression through inhibitory cytokine secretion and in many other ways; they thus play important roles in the TME [68]. Mounting evidence indicates that circRNAs derived from tumor cells can induce immune escape by regulating Treg cell function. For example, circ_0069313 induces immune escape via the miR-325-3p–Foxp3 axis in Treg cells. Consistently, CD8 effector T cells were less infiltrative in oral squamous cell carcinoma (OSCC) tissues with high circ_0069313 expression. Treatment with OSCC cell–derived exosomes increased circ_0069313 and PD-L1 expression in Treg cells. Circ_0069313 depletion inhibited PD-L1 and CD25 expression in OSCC cells, whereas its ectopic expression increased the expression of CD25, but not PD-L1 [69]. These findings indicate that circ_0069313 modulates Treg cell activity. A recent study showed that circGSE1 not only promotes Treg cell function, but also expands the Treg cell population by regulating the miR-324-5p–transforming growth factor (TGF)-β receptor 1–Smad3 axis in hepatocellular carcinoma (HCC). The Treg: CD8^+^ T cell ratio is increased when T cells are cultured with HCC-derived exosomes [70]. In addition, circ_0136666 was shown to activate Treg cells by targeting the miR-497–Akt–mammalian target of rapamycin signaling pathway, to reduce the forkhead box P3 (FOXP3)^+^:CD8^+^ T cell ratio, and to increase the FOXP3^+^:CD4^+^ and FOXP3^+^: CD25^+^ T cell ratios [43].

These findings indicate that circRNAs inhibit antitumor effector T cells and promote Treg-cell expansion and activity via miRNA sponging (Fig. 2). As we described as above, the regulation of circRNAs on T cells and tumor cells is the activation of PD1 / PDL1 signaling pathway. It also indicated that circRNAs are promising potential targets in cancer immunotherapy [71]. However, the binding of PD1 and PDL1 not only reduces the viability and proliferation ability of T cells, but also affects the treatment of immune checkpoint inhibitors in tumors. In addition, the tumor microenvironment of different cancers of different patients are heterogeneous, the roles of circRNAs in cancer immunotherapy is also complicated. Given the important roles of circRNAs in the regulation of T-cell functions, thorough assessment of whether circRNAs regulate the TME in vivo and the translation of these findings into applicable clinical practice would be of interest.

## CircRNAs regulate natural killer cells

Natural killer (NK) cells are composed mainly of T-cell receptor^–^, B lymphocyte antigen receptor^–^, CD56^+^, CD16^+^ lymphoid cells that spontaneously kill tumor cells. Their function depends mainly on the balance between their surface activating receptor natural killer group 2 member D (NKG2D) and the inhibitory killer immunoglobulin-like receptor (KIR). The binding of KIR on NK cells to MHC molecules on a tumor cell inhibits the killing function, whereas activation of the NKG2D ligand on NK cells promotes this function [72].

NK cells kill tumor cells and mediate cytotoxicity mainly by secreting perforin and granzymes. They also express death receptors that mediate the apoptosis of target cells. Increased CD16 and chemokine receptor-3 expression allows NK cells to accumulate and kill cells expressing the chemokine ligand. NK cell dysfunction has been reported to play crucial roles in tumorigenesis and cancer progression [73]. Emerging evidence indicates that circRNAs induce NK cell disability and exhaustion in the TME (Table 2). Various circRNAs, including circUHRF1, circARSP91, circ_0007456, and circ_0048674, play different roles in NK cell regulation via signaling pathways, contributing to the development of cancers including HCC, renal cell carcinoma, and pancreatic cancer (Table 2). In human HCC tissue, high expression levels of circUHRF1 (circ0048677), which originates from ubiquitin-like containing PHD and RING finger domains 1 (UHRF1), are associated with poor clinical prognosis and NK cell dysfunction. Mechanistically, circUHRF1 inhibits NK cell–derived IFN-γ and tumor necrosis factor (TNF)-α secretion and decreases the proportion and tumor infiltration of NK cells by regulating the miR-449c-5p–TIM-3 axis. TIM-3 upregulation induces NK cell exhaustion and promotes HCC progression [74]. Circ_0048674, which also originates from UHRF1, facilitates HCC progression and NK cell exhaustion through a different mechanism; it serves as an miR-223-3p sponge to alter PD-L1 expression. Circ_0048674 knockdown inhibits tumor cell proliferation, migration, and apoptosis and impairs NK cell function [75]. Whether these circRNAs work cooperatively or competitively to support tumor immune evasion remains largely unknown.

**Table 2:** circRNAs regulate NK cells.

circRNA	Targets/pathways	regulation of immune responses	Cancer	References
circUHRF1	KLF16/circUHRF1/ sponging miR-449c-5p/Tim-3	suppress NK cell secretion of IFN-γ and TNF-α	hepatocellular carcinoma	[74]
Hsa_circ_0048674	Hsa_circ_0048674 /miR-223-3p/PDL1	inhibit the functions and promotes NK cells exhaustion	hepatocellular carcinoma	[75]
CircARSP91	CircARSP91/ULBP1 in HCC	enhances the cytotoxicity of NK cells	hepatocellular carcinoma	[76]
Hsa_circ_0007456	Hsa_circ_0007456/miR-6852-3p/ICAM-1 axis	regulated the susceptibility of HCC to NK cells	hepatocellular carcinoma	[77]
circ_0000977	circ_0000977/miR-153 axis modulates HIF1A-mediated immune escape	inhibit the killing effect of NK cells on pancreatic cancer tumor cells	pancreatic carcinoma	[80]
circZKSCAN1	KLF2-induced circZKSCAN1/miR-1294/PIM1 axis	attenuate NK cell-mediated cytotoxicity	renal cell carcinoma	[81]
circFOXO3	circFOXO3/miR-29a-3p and miR-122-5p	attenuate NK Cell-Mediated Cytotoxicity	renal cell carcinoma	[85]
circ_0001005	AR/circ_0001005/PD-L1 axis in impacting NK cell antitumor efficacy	damage the function of NK cell antitumor efficacy	urinary bladder cancer	[87]

In contrast to the role of circRNAs in suppressing NK cells described as above, mounting evidence has indicated that some circRNAs enhance the cytotoxicity of NK cells and inhibit the malignancy of HCC. CircARSP91 was reported to coordinate with tumor suppressors to exert anti-HCC effects. Specifically, it boosts the expression of UL16 binding protein 1, which binds to the NKG2D ligand on NK cell surfaces, mediating NK cell activation and cytotoxicity [76]. Similarly, circ_0007456 influences HCC susceptibility to NK cells by enhancing intercellular cell adhesion molecule-1 (ICAM-1) expression through miR-6852-3p sponging [77]. ICAM-1, also called CD54, is a member of the immunoglobulin superfamily of adhesion molecules that plays a crucial role in adhesion reaction mediation. Recent studies show that ICAM-1 on tumor exosome surfaces mediates the adhesion of the exosomes to CD8^+^ T cells, which is prerequisite for PDL1–mediated immunosuppressive effects [78, 79]. Accordingly, ICAM-1 may function as an essential checkpoint or potential therapeutic target downstream of circ_0007456 in the setting of HCC.

**Fig. 3:**
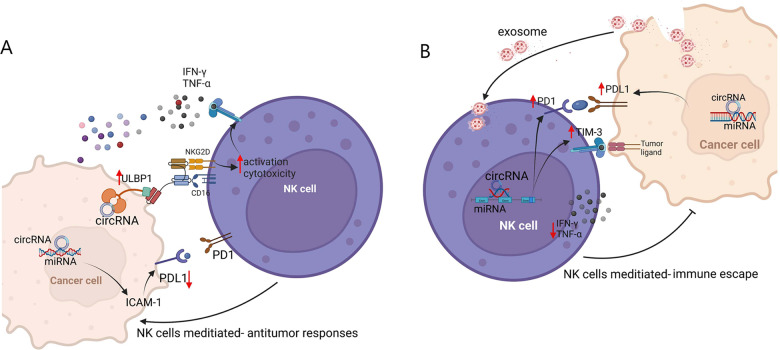
Circular RNAs have bidirectional modulatory effect on NK cells. **A** Circular RNAs promoting NK-mediated antitumor responses. **B** Circular RNAs suppressing NK cell activity to trigger tumor immune escape. ULBP1 human UL 16-binding protein 1, ICAM-1 intercellular cell adhesion molecule-1, NKG2D natural killer group 2 member D, TIM-3 T cell immunoglobulin and mucin domain-containing protein 3.

CircRNAs have also been reported to regulate the NK cell immune response against many other cancer types. For example, circ_0000977 was reported to be upregulated in pancreatic cancer cells under hypoxia and to induce tumor immune escape via the miR-153–hypoxia inducible factor 1α–a disintegrin and metalloprotease (ADAM) 10 axis. ADAM10 upregulation prompted membrane major histocompatibility complex class I chain-related gene A (MICA) shedding from pancreatic ductal adenocarcinoma cell surfaces and conversion to soluble MICA to degrade NKG2D on NK cells. This reduction of NKG2D expression resulted in NK cell hyporesponsiveness, and thus the inactivation of innate and adaptive immune responses and escape from immune surveillance [80]. CircZKSCAN1 is generally upregulated in clear cell renal cell carcinoma (ccRCC), and its downregulation significantly enhanced NK cell–mediated toxicity to RCC cells. It was found to modulate proviral integration site for Moloney murine leukemia virus-1 (PIM1) expression to inhibit NK cell–mediated toxicity to ccRCC cells via miR-1294 sponging [81]. However, the exact molecular mechanism underlying the role of PIM1 in NK cells remains unknown. Kruppel-like factor (KLF) is a transcription factor with a zinc finger structure that participates in the regulation of gene transcription, which is related to cell proliferation and differentiation and tumorigenesis [82, 83]. KLF2 inhibits early-stage NK cell proliferation and maintains a static late-stage NK cell state [84]. In addition, KLF16 has been shown to have an important role in suppressing NK cell–mediated cytotoxicity. It transcriptionally activates circFOXO3, which sponges miR-29a-3p and miR-122-5p to aggravate NK cell toxicity to ccRCC cells [85]. The androgen receptor, an oncogene, is associated closely with invasion and drug resistance in different cancers, including prostate cancer (PCa) and BCa [86]. It has been reported to upregulate circ_0001005 expression, attenuating NK cell killing efficacy by affecting PD-L1 expression via miR-200a-3p sequestration in BCa [87]. In addition, circRHOT1 upregulation has been found in BCa and is associated with the attenuation of NK cell–mediated toxicity to BCa cells. ZNF652, a member of the largest family of transcription factors that plays roles in the proliferation, invasion, and metastasis of many cancer types, induces circRHOT1 expression [88–90]. Although many circRNAs serve as miRNA sponges, some regulate the antitumor toxicity of NK cells through distinct mechanisms. circARSP91 was recently reported to bind directly to tumor suppressors to exert anti-HCC effects [91]. Thus, circRNAs can act as pleiotropic TME modulators by regulating tumor and NK cells (Fig. 3).

## CircRNAs regulate macrophages

Macrophages are major lymphocytes that infiltrate solid tumors. Those infiltrating tumor tissues or distributed in the TME are called tumor-associated macrophages (TAMs) and have a central role in initiating the innate immune response, which leads to activation of the adaptive response in the later phase. Macrophages present two polarized states: classical (M1) and alternative (M2) activation, which occur through distinct pathways during mature differentiation. M1 TAMs are induced by IFN-γ, granulocyte-macrophage colony-stimulating factor (CSF), TNF-α, and many other cytokines and are able to kill tumor cells, whereas M2 TAMs are activated mainly by interleukin (IL)-4, IL-13, TGF-β, macrophage CSF, and other cytokines to promote tumor progression by activating a type 2 helper T cell–type immune response [92]. In addition to causing immunosuppression, M2 TAMs promote tumor growth and metastasis through many other mechanisms, such as tumor invasion, leakage to blood vessels, and angiogenesis promotion [93]. Most tumors do not have M1 macrophages without specific antigens and other factors. Thus, most TAMs have the M2, which promotes tumor occurrence, development, and metastasis, although they have the potential to repolarize to M1 macrophages [94].

Some circRNAs have been reported to regulate macrophage polarization in many cancers. CircTMEM181 prompts T-cell exhaustion by sponging miR-4883p to upregulate CD39 expression in macrophages, indicating that it affects mainly the macrophages in the immune microenvironment, rather than HCC cells [95]. Consistently, elevated circTMEM181 expression is correlated with anti–PD-1 treatment resistance and poor prognosis in patients with HCC. As another example, circ_0110102 upregulates C–C motif chemokine ligand (CCL) 2 expression by inhibiting miR-580-5p in HCC. CCL2 then activates the cyclooxygenase-2/prostaglandin E2 pathway in macrophages via FoxO1 in a p38 mitogen-activated protein kinase–dependent manner [96]. Tumor cells can recruit macrophages into tumor tissue through the secretion of many chemokines; tumor cells then secrete various cytokines, metabolites, and exosomes to alter and polarize the function of TAMs. Circ_0003410 was shown to promote HCC cell proliferation and migration via miR-139-3p sponging and thus the upregulation of CCL5 expression, which recruits M2 macrophages to enhance HCC deterioration in vitro *and* in vivo [97]. Similarly, circ_0074854 was shown to inhibit HCC tumorigenesis, mainly through the suppression of M2 macrophage polarization in vitro *and* in vivo [98]. However, the mechanism underlying this polarization regulation needs to be investigated further. CircASAP1 was found to regulate the expression of CSF-1, which controls the macrophage production, differentiation, and function [99], through the miR326/ miR-532-5p–CSF-1 signaling pathway, resulting in CD68^+^ TAM infiltration and HCC growth and metastasis [91].

Many reports describe circRNA regulation of macrophages in the microenvironments of tumors other than HCC, such as esophageal squamous cell carcinoma (ESCC), non-small cell lung cancer (NSCLC), and PCa. Different circRNAs affect TAMs through different pathways or regulators. For example, circRNA TCFL5 promotes esophageal cancer progression by regulating M2 macrophage polarization via the miR-543–formin-like protein 2 axis [100]. Similarly, circ-0048117 upregulates toll-like receptor 4 expression via miR-140 sponging to promote M2 macrophage polarization, prompting ESCC invasion and metastasis [101]. CircPLCE1 and Circ_0006990 have been demonstrated to motivate TAM M2 polarization in the TME through the miR-485-5p–actin-γ1 and miR-132-3p–mucin 13 cell surface associated axes, respectively, in CRC [102, 103]. A recent study showed that the flavonoid quercetin significantly reversed the promotion of M2-TAMS on proliferation of CRC cell by downregulating circ_0006990 [103]. CircSHKBP1 and circFARSA promote NSCLC migration and invasion by inducing M2 macrophage polarization and impairing CD8^+^ T cell function in vitro *and* in vivo [104, 105]. Similarly, Gao et al have demonstrated that exosomal circZNF451 could induce M2 polarization of macrophages and exhaustion of cytotoxic CD8 + T cells to reshape the TME via the FXR1- ELF4-IRF4 axis. More importantly, they will limit the sensitivity of anti-PD1 treatment in vitro *and* in vivo (or in C57BL/6 J mice) [106]. Myeloid-derived suppressor cells (MDSCs) are bone marrow–derived immature cells that suppress T cells and are activated and mobilized under pathological conditions, such as cancer. Their main functions are to promote tumor development and tumor-related TAM transformation [107, 108]. In lung cancer, circPTK2 and circHIPK3 play important roles in monocytic MDSC differentiation into CD163^+^ M2 macrophages [109].

Cytokines and chemokines play crucial roles in M2 macrophage polarization. CircSMARCC1 has been shown to regulate CCL20 expression by suppressing miR-1322 activity, thereby mediating M2 macrophage polarization and infiltration, in PCa [110]. In breast cancer cells, circWWC3 upregulates IL-4 expression and secretion to induce M2 macrophage polarization [111] and T-cell inactivation, leading to immune escape [112]. In addition, CircITGB6, circsafb2, and circNEIL3 promote M2 macrophage polarization in ovarian cancer, kidney cancer, and gliomas, respectively [113–115].

**Fig. 4:**
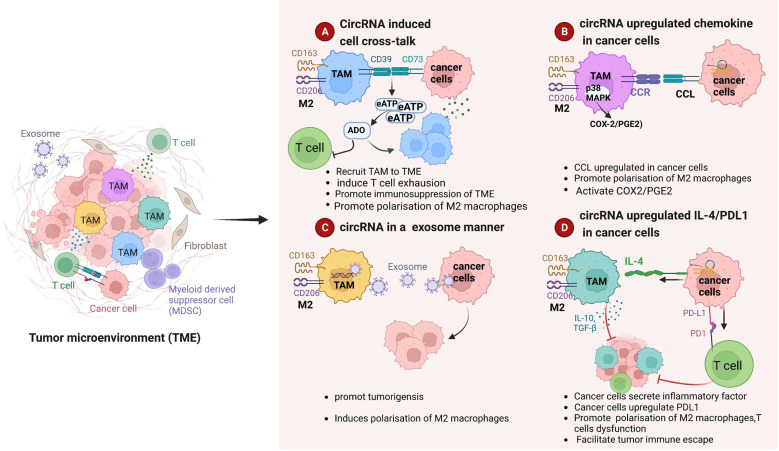
Circular RNAs (circRNAs) regulate macrophage polarization to promote tumor progression. **A** CircRNAs mediate crosstalk between tumor cells and macrophages, induce M2 macrophage polarization, and impair T cell function, resulting in the formation of an immunosuppressive tumor microenvironment (TME). **B** CircRNAs upregulate chemokine expression in tumor cells and induce M2 macrophage polarization. **C** Tumor cells release exosomes containing circRNAs into macrophages to enhance M2 macrophage polarization. **D** CircRNAs promote the secretion of inflammatory factors and immunosuppressive molecules in tumor cells to recruit and induce M2 macrophage polarization and disable T cells.

TAMs form the most abundant immune cell population in the TME. CircRNAs in tumor cells can regulate macrophage polarization through multiple pathways in the TME (Table 3, Fig. 4): (1) they induce crosstalk between tumor cells and macrophages (Fig. 4A), (2) they promote chemokine secretion from tumor cells (Fig. 4B), (3) tumor cell-derived circRNAs in exosomes enter macrophages to play a regulatory role (Fig. 4C), and (4) they promote tumor-cell expression of cytokines such as IL-4 and PD-L1 (Fig. 4D). In-depth investigation of the mechanisms underlying these roles and preclinical studies are urgently needed.

**Table 3:** circRNAs regulate .macrophages.

circRNA	Targets/pathways	regulation of immune responses	Cancer	References
CircASAP1	CircASAP1/ miR326/miR-532-5p-CSF-1/	promotes TAM infiltration	hepatocellular carcinoma	[91]
hsa_circ_0001663 (circTMEM181)	circTMEM181/ sponging with miR-488-3/ inducingCD39 expression in macrophages	induces macrophage M2-like polarization	hepatocellular carcinoma	[95]
hsa_circ_0110102	circ_0110102/miR-580-5p/PPARα/CCL2	inhibits the pro-inflammatory cytokine release from macrophages	hepatocellular carcinoma	[96]
hsa_circ_0003410	hsa_circ_0003410/miR-139-3p/CCL5 axis/increasing the ratio of M2/M1 macrophages	increases the ratio of M2/M1 macrophages	hepatocellular carcinoma	[97]
hsa_circ_0074854	hsa_circ_00074854 / macrophage M2 polarization/immune escape	promotes Macrophage M2 Polarization	hepatocellular carcinoma	[98]
circ TCFL5	circRNA TCFL5 /miR-543-FMNL2 axis / modulating M2 macrophage polarization	modulates M2 macrophage polarization	esophageal squamous cell carcinoma	[100]
circ0048117	hypoxic/exosomal-circ0048117/miR-140 / TLR4/promoting M2 polarization	promoting M2 polarization	esophageal squamous cell carcinoma	[101]
hsa_circ_0006990	circ_0006990/miR-132-3p/MUC13 axis/promotes the polarization of M2 macrophages	promotes the polarization of M2 macrophages	colorectal cancer	[103]
CircPLCE1	Circ PLCE1/miR485-5p/ACTG1 axis/ modulates TAM M2 polarization	modulates TAM M2 polarization	colorectal cancer	[102]
circFARSA	exosomal-circFARSA /PTEN/PI3K/AKT /mediates M2 macrophage polarization	promotes M2 polarization	non-small cell lung cancer	[105]
circSHKBP1	circSHKBP1/promoting M2 polarization and macrophage recruitment.	promotes M2 polarization and macrophage recruitment	non-small cell lung cancer	[104]
circHIPK3	circHIPK3/PTK2/infiltration of M2 polarization		lung cancer	[109]
CircSMARCC1	CircSMARCC1/miR-1322/CCL20/CCR6/inducing TAMs infltration and M2 polarization	induces M2 macrophage polarization	prostate cancer	[110]
circWWC3	circWWC3/ IL-4, PD-L1/ M2-like TAM polarization	modulates M2 macrophage polarization	breast cancer	[112]
circITGB6	circITGB6 / FGF9/ induces polarization of M2 macrophages	induces polarization of TAMs toward M2 phenotype	Ovarian cancer	[113]
circSAFB2	circSAFB2 /miR-620/JAK1/ STAT3 axis /inducing polarization of M2 macrophages	induces polarization of M2 macrophages	renal cell carcinoma	[114]
circNEIL3	exosomes-EWSR1 / circNEIL3/ stabilizing IGF2BP3/macrophage immunosuppressive polarization	Macrophage immunosuppressive polarization	glioma	[115]

## CircRNAs regulate neutrophils, myeloid-derived suppressor cells, and cancer-associated fibroblast

The neutrophils are also an important component in the TME, participating in different stages of tumor development and progression such as tumorigenesis, proliferation and metastasis. Neutrophils could play dual roles as a pro-tumor(N2) or tumor suppressor (N1) in the tumor microenvironment due to heterogeneous phenotypes and functional diversity. Recently, mounting evidence show that circular RNA affects tumor development by regulating the function of neutrophils. In bladder cancer, circDHTKD1 recruited and activated neutrophils by inducing CXCL5 expression, and then neutrophils participated in lymphangiogenesis by secreting VEGF-C, facilitating lymphatic metastasis of bladder cancer cells [116]. But in CRC, circPACRGL mainly promoted differentiation of N1 to N2 neutrophils by sponging miR-142-3p/miR-506-3p, N2 neutrophils increased the expression of transforming growth factor-β1 (TGF-β1), which promoted CRC cell proliferation, migration and invasion [117]. Although the underlying mechanism is not very clear, but the diversity and plasticity of neutrophils maybe act as a potential and promising immunotherapy target in clinical treatment.

Myeloid-derived suppressor cells (MDSC) are also another key player in TME. In addition to the immunosuppressive effect, MDSC can also exert tumor-promoting effects by promoting angiogenesis, invasion and metastasis. More details about non-coding RNAs including circRNAs modulate MDSCs in TME have been summarized elsewhere [118].

Cancer-associated fibroblasts (CAFs), also named as tumor-associated fibroblast, are a key factor in tumor microenvironment. It plays important role in tumor growth and metastasis due to diverse functions, such as interactions with cancer cells and crosstalk with infiltrating leukocytes and so on. In pancreatic cancer, Hu et al found that circFARP1 upregulated the expression and secretion of LIF via CAV1 in CAFs to induce chemoresistance [119]. In addition, in other cancers, circRNAs derived from CAFs also can promote tumor progression. For example, circEIF3K from CAF promotes CRC progression *via* miR-214/PD-L1 axis [120]. Exosomal circSLC7A6 from CAF promote tumorigenesis of CRC by regulating CXCR5 [121]. CAF-derived CXCL11 modulates HCC migration and metastasis through the circUBAP2/miR-4756/IFIT1/3 axis [122]. All evidence suggesting an oncogenic role of CAFs in tumorigenesis and indicating CAFs or circRNAs can considered as potential target in immunotherapy.

## Conclusion and perspectives

For several decades following the discovery of RNA viruses in 1976 and eukaryotes in 1979 [1, 123], circRNAs were considered to be splicing errors. With the rapid development of RNA sequencing technologies and bioinformatics, numerous circRNAs have been identified and their roles in various diseases, especially cancer, have been investigated extensively. As reviewed here, circRNAs form a multifaceted class of regulators that play multiple roles in tumorigenesis, tumor progression and metastasis, and treatment resistance. They can act as miRNA sponges or interact with RBPs. Different circRNAs may regulate the same downstream gene expression by sequestering different miRNAs; for instance, circ_0046523 induces PD-L1 expression via miR-148a-3p sponging in pancreatic cancer [48], whereas circKRT1 induces PD-L1 expression via miR-495-3p sponging in OSCC [54]. However, our current knowledge of circRNA functions has been obtained mainly from cell-based studies. The examination of whether circRNA loss regulates tumor immune responses and cancer development in genetic mouse models would be of great interest. Despite much progress in past decades, the establishment of circRNA nomenclature rules remains a pressing issue. The production of different circRNAs from the same gene due to alternative splicing can cause confusion. Additionally, the molecular mechanisms underlying this process and the different roles of these circRNAs need to be elucidated.

The TME is a complex integrated system containing tumor cells, tumor-infiltrated immune cells, blood vessels, extracellular matrix, and signaling molecules. Accumulating evidence has revealed that circRNAs play crucial roles in TME regulation, such as tumor immune evasion, metastasis, and metabolism. However, the precise physiological and pathological roles of circRNAs in the TME and related underlying mechanisms remain largely unclear. In this review, we have described the roles of circRNAs in the TME, especially in TME-related immune cells such as T cells, NK cells, and macrophages. CircRNA in the TME can upregulate the expression of the immune checkpoint molecules PD-L1, PD-1, and CD73 on tumor cell surfaces via miRNA sponging, helping tumor cells to escape recognition and death by T cells [51–53, 57]. Stromal cells, such as cancer-associated fibroblasts, endothelial cells, and pericytes, are important TME components, and much more research is warranted to explore their potential regulation. Although TME reprogramming is considered to be a potentially effective strategy for tumor eradication and the improvement of tumor immunotherapy efficacy, there is still a long way to go before we can conquer cancer. For example, does dose targeting of circRNAs work in mouse models, at least in three-dimensional tumor organoid models? Is circRNA targeting applicable in clinical trials? Thorough investigations of circRNAs using animal models would help to accelerate the translation of basic research into clinical practice. We believe that an improved understanding of circRNA functions and mechanisms related to tumorigenesis and immunotherapy would certainly contribute to the development of new therapeutic strategies for cancer.
